# Dual roles of methoprene-tolerant gene *TaMet* in male molting and female reproduction of the tomato leafminer, *Tuta absoluta* (meyrick)

**DOI:** 10.3389/fphys.2024.1500391

**Published:** 2024-11-14

**Authors:** Tingwei Zhang, Kai Xu, Deqian Liu, Hang Ma, Wenbiao Liu, Wenjia Yang

**Affiliations:** ^1^ Key Laboratory of Surveillance and Management of Invasive Alien Species in Guizhou Education Department, College of Biological and Environmental Engineering, Guiyang University, Guiyang, China; ^2^ Yunnan Yuantianhua Co., Ltd Research and Development Center, Kunming, China

**Keywords:** invasive species, Tuta absoluta, RNAi, functional differentiation, reproduction, molting

## Abstract

The tomato leafminer (*Tuta absoluta*) is a highly destructive global quarantine pest. The methoprene-tolerant (Met) protein, a member of the bHLH/PAS family of transcription factors, forms complexes with other family members to transduce the juvenile hormone signal, which regulates insect growth and development. However, the functions of the *TaMet* gene have rarely been studied in *T. absoluta*. Herein, we investigated the significance of *TaMet* in *T. absoluta*. Spatiotemporal expression analysis revealed that *TaMet* exhibited comparable expression patterns in males and females, with high expression levels during the early pupal and early adult stages. *TaMet* was predominantly expressed in the female ovary and male wing. *TaMet* knockdown impaired ovarian development in female adults, causing irregular arrangement and increased spacing of the egg epithelial cells in the ovary. Silencing *TaMet* also led to a 67.25% reduction in female spawning and a 67.21% decrease in the offspring hatching rate. Furthermore, the vitellogenin content was significantly diminished, and the expression levels of vitellogenin (*Vg*) and vitellogenin receptor (*VgR*) genes were significantly downregulated. In contrast, silencing *TaMet* in 3-day-old male pupae resulted in an 80% mortality rate and various phenotypic abnormalities, including body melanism, molting defects, and wing deformities. Moreover, the expression levels of wing development and chitin metabolism genes decreased significantly after knocking down *TaMet.* Our results indicate that *TaMet* plays a significant dual role in male molting and female reproduction of *T. absoluta*.

## 1 Introduction

In holometabolous insects, precise hormone regulation facilitates development from eggs to sexually mature adults through several molts, thereby controlling population dynamics (Liu et al., 2018; Smykal et al., 2014). Juvenile hormone (JH), a structurally unique sesquiterpenoid hormone, influences metamorphic development in larvae/nymphs by antagonizing the ecdysone-signaling pathways (Mukai et al., 2022). In adults, JH predominantly functions as a gonadotropin, promoting reproduction by regulating various physiological and behavioral processes in both males and females. *Bactrocera dorsalis* larvae exhibit premature pupation when there is insufficient JH, significantly compromising pupal survival (Liu et al., 2017). In *Aedes aegypti*, the JH signal is crucial for regulating ribosomal protein synthesis, i.e., *RPL32* and *RRS*1, and facilitates vitellogenin (*Vg*) synthesis in the fat body (Wang et al., 2017). JH also induces the expression of genes associated with *Vg* uptake, such as vitellogenin receptor (*VgR*) and lipophorin receptor (*LpR*), thereby promoting ovarian development (Marchal et al., 2014; Parra-Peralbo and Culi, 2015; Ahmed et al., 2022). Thus, the JH signaling pathway has emerged as a prominent area of investigation in entomological research.

Regardless of its chemical structure, form, or function, most regulatory effects of JH in insects are mediated by its receptors. The receptor *Methoprene-tolerant* (*Met*) and its partner *Taiman* (*Tai*), also known as *βFtz-F1* steroid hormone receptor coactivator (FISC)/steroid receptor coactivator (SRC), are central to JH signaling, together influencing insect growth and development (Zhang et al., 2011). However, vitellogenesis and oocyte maturation in insects are regulated by JH receptor complexes through different pathways. JH-Met-Tai promotes *Vg* expression via insulin-like peptides (*ILPs*) in *Tribolium castaneum* (Li et al., 2011; Zhang et al., 2023). The JH receptor complex directly activates the expression of *Krüppel-homolog 1* (*Kr-h1*) and *Hairy* in mosquitoes to facilitate post-eclosion ovarian maturation (Saha et al., 2019). Additionally, JH and 20-hydroxyecdysone (20E) are key hormones regulating insect molting and metamorphosis. In the *Bombyx mori Met1* knockout strain, 20E signaling is significantly activated in the larval epidermis, leading to early epidermal development and advanced pupation (Daimon et al., 2015). The *Broad* gene, an early responder in the 20E signaling pathway, is regulated by JH signaling genes, such as *Kr-h1*, which affect metamorphosis (Minakuchi et al., 2009).

Met was initially identified as a transcription factor having a basic helix-loop-helix Per/Arnt/Sim (bHLH-PAS) structure and directly binding to JH via the PAS-B domain, forming JH-Met, in *Drosophila melanogaster* (Ashok et al., 1998). This complex enters the nucleus using heat shock proteins. JH-Met forms a transcription complex in the nucleus with Tai/FISC, targeting downstream gene promoters at the Ebox element, thereby inducing gene expression and activating the JH signaling cascade. Insect *Met* is essential in various physiological processes, including reproductive development, metamorphosis, and toxin response (Julide et al., 2013; Lozano and Belles, 2014). RNA interference (RNAi)-mediated silencing of *Met* significantly reduced *Vg* and *VgR* expression, causing abnormal ovarian morphology and decreased egg production and hatchability in *Neoseiulus barkeri* females (Wu et al., 2021). The JH receptor, Met, and its target genes, *Kr-h1* and *Broad-Complex* (*BR-C*), are crucial for metamorphosis in holometabolous insects. In *A. aegypti*, knockout the *Met* gene decreased the expression of *Kh-h1* and caused severe molt block in larval–pupal transition (Zhu et al., 2019). Met1 phosphorylation via prokaryotic expression enhances the binding of the Met1-Tai complex to the JHRE E-box, thereby regulating *Kr-h1* transcription in *Helicoverpa armigera* (Li et al., 2021). *Met* deficiency significantly boosts *BR-C* expression in *B. mori* larvae, leading to early metamorphosis and deformed adult wings (Hu et al., 2019). *Met* binds to the E-box in the diptericin gene’s regulatory region in *A*. *aegypti* mosquitoes, indicating that JH suppresses *diptericin* (*Dpt*) gene expression via the *Met* receptor (Chang et al., 2021).

The tomato leafminer *Tuta absoluta* (Meyrick) is an important invasive Lepidopteran pest that arrived from North America to Xinjiang in 2007 and rapidly spread across China, including Sichuan, Shandong, and Tianjin (Biondi et al., 2017). Larvae damage plants by eating leaves and penetrating leaf veins, leading to yellowing, wilting, and up to 80% crop loss (Uulu et al., 2017). Chemical pesticides, such as pyrethroids and avermectin, and some fungicides are effective for rapidly controlling *T. absoluta*. The large and widespread use of these pesticides hass inevitably resulted in varying resistance in *T. absoluta* (Cherif et al., 2017; Guedes et al., 2019; Siqueira et al., 2000). In Brazil and Chile, *T. absoluta* has developed resistance to many insecticides, making it necessary to identify new green approaches for tomato leaf miner control. *Met* is known to play critical roles in insect growth and development, but specific information about its functions in *T. absoluta* remains limited despite some studies having cloned the *TaKr-h1* and *TaMet* genes (Wang et al., 2023). Focusing on insect reproduction and molting, the RNAi-based functional study of Met in *T. absoluta* may enhance pest control strategies and potentially replace traditional chemical pesticides.

## 2 Materials and methods

### 2.1 Insects

The tested *T. absoluta* population was originally collected from Kunming City, Yunnan Province, China, in 2023. The insects were reared in the sunlight insectary room of Guiyang University under the following conditions: temperature 27°C ± 1°C, relative humidity 55% ± 5%, and light cycle 16 L: 8 D. The larvae were used to inoculate fresh tomato plant leaves. Each cage is equipped with 15% honey water for adults.

### 2.2 Developmental and tissue-specific expression analysis

The same batch of hatched larvae were used to inoculate the leaves of tomato seedlings, and *T. absoluta* samples were collected at different developmental stages (1–7 days for male and female pupae, and 1–2 days for adults). Insects were collected as one sample at each developmental stage, and three biological replicates were prepared. Nine tissues, including the head, epidermis, intestinal tract, Malpighian tubule, fat body, ovary, seminal vesicle, male wing, and female wing, were dissected from day-2 adults, with 50 insects in each sample and three biological replicates. Total RNA was extracted from the samples using TransZol reagent (TransGen Biotech, Beijing, China). Subsequently, the first-strand cDNA was synthesized using the PrimeScriptTM RT Reagent Kit (TaKaRa, Tokyo, Japan), according to the manufacturer’s instructions. qPCR was used to investigate the expression profiles of *TaMet*. The qPCR primers were designed using primer3 (https://primer3.ut.ee). The qPCR mix included 10 μL of TransStart®  Top Green qPCR SuperMix (TransGen Biotech), 7 μL of ddH_2_O, 1 μL of the cDNA template, and 1 μL each of the forward and reverse primers (10 μmol/L). The qPCR reaction procedure was performed as follows: predenaturation at 95°C for 5 min and 40 cycles of denaturation at 95°C for 15 s, annealing at 60°C for 30 s, and extension at 72°C for 30 s. The melting curve was analyzed at 60–95°C. The *EF1α* gene (GenBank: MZ054826) of *T. absoluta* was used as the reference gene (Yan et al., 2021). The relative expression levels of *TaMet* were calculated using the 2^−ΔΔCT^ method (Livak and Schmittgen, 2001).

### 2.3 Effects of *TaMet* RNAi on ovarian development and female reproduction

Specific primers containing the T7 RNA polymerase promoter sequence were designed using Primer 5.0 (Supplementary Table S1), and double-stranded RNA (dsRNA) was synthesized using the TranscriptAid T7 High Yield Transcription Kit (Thermo Fisher Scientific, America). After purification, the synthesized dsRNA was diluted to 1 μg/μL with RNAi injection buffer. The same-aged female insects were collected for microinjection at the pupal stage (3-day-old), and samples were collected at different times (24, 48, and 72 h) for total RNA isolation. The RNAi silencing efficiency was evaluated using qPCR, as described above. Two-day-old female adults were dissected and photographed with a stereomicroscope VHX-2000C (Keyence Corporation, Osaka, Japan), and their ovaries were graded according to the previously published ovarian classification (Yang et al., 2024). After their emergence, female adults were reared with same-aged male insects in a 1:3 ratio in a new cage. Egg production and hatching were recorded within 10 days, and the changes in the ovarian morphology of the females were dissected and observed by measuring the ovarian tube length and egg size. Similarly, qPCR was used to detect the mRNA levels of key genes (*TaVg* and *TaVgR*) that may affect reproduction. Another batch of 3-day-old female pupae was also injected with ds*TaMet*, and the samples were frozen with liquid nitrogen after 24 h. The vitellogenin content was measured using the Insect Vitellogenin Enzyme-linked immunosorbent assay (ELISA) Kit (Mlbio, Shanghai, China).

### 2.4 Phenol fuchsin stain

The ovarioles from *T. absoluta* females were dissected in phosphate-buffered saline (PBS) and fixed in 4% paraformaldehyde fixative. The ovarioles were randomly selected from the ds*TaMe*t- and ds*GFP*-injected groups, rinsed 3 times with PBS, stained with improved phenol fuchsin stain (Yuanye, Shanghai, China) for 15 min at room temperature, and squashed under a coverslip. All images were captured using an LSM 900 confocal laser-scanning microscope (Zeiss, Oberkochen, Germany).

### 2.5 Effect of *TaMet* RNAi on male molting and wing development

Three-day-old male pupae were collected for dsRNA injection, and the mortality rate and the death phenotype were recorded for 20 days. The expression of four wing development genes, including *TaWG* (*wing less*), *TaAP* (*apterous*), *TaSRF* (*serum response*), and *TaVG* (*vestigial*), four chitin synthesis genes including *TaChs* (*chitin synthase*), *TaTre1* (*trehalase 1*), *TaTre2* (*trehalase 2*), and *TaUAP* (*UDP-N-acetylglucosamine pyrophosphorylase*), and five chitin degradation genes including *TaCDA1* (*chitin deacetylase 1*), *TaCDA2* (*chitin deacetylase 2*), *TaCht5* (*chitinase 5*), *TaCht7* (*chitinase 7*), and *TaCht10* (*chitinase 10*) were detected using qPCR.

### 2.6 Statistical analysis

Statistical analyses were performed using SPSS 20.0 software (IBM, Chicago, IL, USA), and the data were visualized using GraphPad Prism 6.01 (GraphPad, La Jolla, CA, USA). The survival rates were analyzed using the Kaplan-Meier method, and the Log-Rank test was used to determine the significant difference between the RNAi-treatment and control groups. Differences between the two groups were compared using a Student’s t*-*test at a significance level of *p* < 0.05. A one-way analysis of variance (ANOVA) was applied to compare the differences among more than two samples.

## 3 Results

### 3.1 Developmental and tissue-specific expression of *TaMet*

**FIGURE 1:**
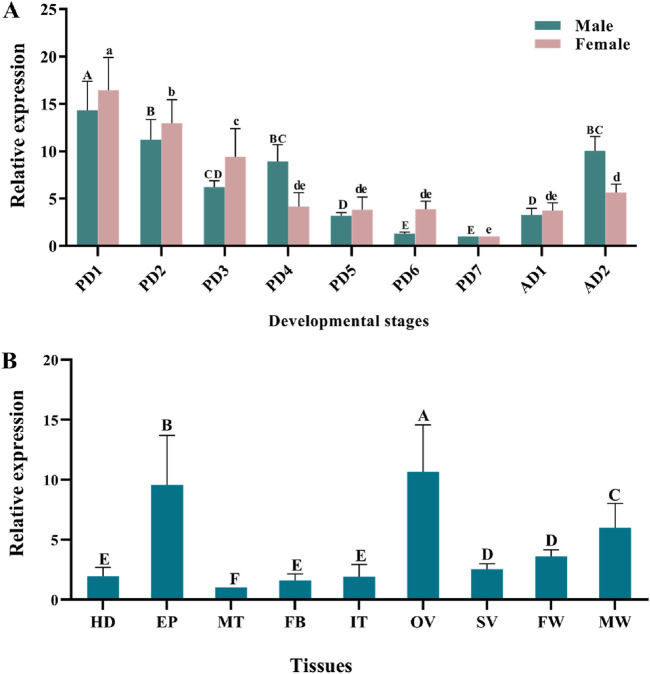
Relative expression of *TaMet* in Tuta absoluta at different developmental stages **(A)** and adult tissues **(B)**. Total RNA was isolated from pools of 1–7-day pupae (PD1-PD7), 1–2-day adults (AD1-AD2), and the tissues of the head (HD), epidermis (EP), intestinal tract (IT), Malpighian tubule (MT), fat body (FB), ovary (OV), seminal vesicle (SV), female wing (FW), and male wing (MW). Different letters above bars indicate significant differences for females or males or the tissues based on one-way ANOVA, followed by a least significant difference test (*p* < 0.05).

Quantitative real-time PCR (qPCR) results showed that *TaMet* was highly expressed during the early pupal and early adult stages in males. Specifically, *TaMet* expression in day-1 male pupae was 12.58-fold higher than that in day-7 male pupae. A similar expression pattern was observed in females, with *TaMet* expression peaking on the first day of pupation and being 16.45-fold higher than on the seventh day (Figure 1A) (*F* = 6.781; *df* = 6; *p* < 0.001). Tissue expression profiling revealed that *TaMet* was highly expressed in the epidermis and ovaries of *T. absoluta*, with 9.55- and 10.65-fold higher expression than in the Malpighian tube, respectively. Besides, *TaMet* was also highly expressed in the female wing and male wing (Figure 1B).

### 3.2 *TaMet* knockdown resulted in abnormal ovarian development

**FIGURE 2:**
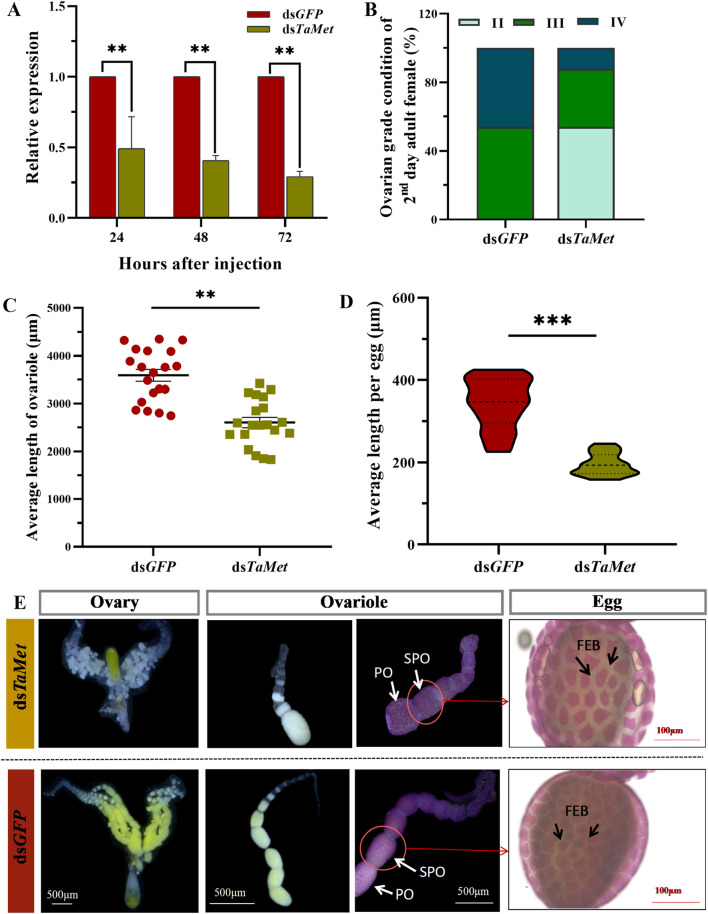
Effect of *TaMet* silencing on ovarian development in *Tuta absoluta* females. **(A)** Relative expression levels of *TaMet* at 24, 48, and 72 h after *TaMet* or *GFP* dsRNA injection. **(B)** Changes in the ovarian grade ratio after silencing *TaMet*. **(C)** Length of ovarian tubules in females after dsRNA injection. **(D)** Average length of egg grains after dsRNA injection. **(E)** Effects of *TaMet* RNAi on the follicular epithelium of primary oocytes. Morphology of the follicular epithelium of primary oocytes in 2-day-old adults. PO, primary oocyte; SPO, sub-primary oocyte; FEB, follicular epithelium between. Significant differences between the treatment and control groups were determined using a Student’s *t*-test (*p < 0.05, **p < 0.01, ***p < 0.001). II: ovarian grade II, yolk deposition stage; III: ovarian grade III, mature and waiting period; IV: ovarian grade IV, peak spawning stage.

*TaMet* expression was significantly suppressed up to 70.8% at 72 h post-dsRNA injection in females (Figure 2A) (*t* = 32.977, *p* < 0.001). The ovaries were mainly at level III and IV (high production period) in the control group (ds*GFP*), while those of the treatment group were significantly delayed. Specifically, the ovarian development levels were multipolar (II, III, and IV were present), with level II ovaries (development period) being dominant (Figure 2B). The average length of the ovarian tube in the ds*TaMet*-injected group was 2601.4 μm, which was 27.5% lower than that of the control group at 3,588.6 μm (Figure 2C) (*t* = 6.017, *p* < 0.01). In addition, the ds*GFP* group had more mature oocytes with a mean length of 343.2 µm, while the ds*TaMet* group had fewer mature oocytes with a mean length of 194.7 µm (Figure 2D) (*t* = 10.172, *p* < 0.001). The ovaries of 2-day-old female adults in the ds*GFP* group were mature and contained many mature egg particles. In contrast, the ovaries of the treatment group were atrophied, and the ovarian tubes contained fewer egg particles. Phenol margin staining showed that the follicular epithelial cells on the surface of the secondary oocytes in the ds*GFP* group were closely packed, with sufficient yolk precipitation. However, follicular epithelial cells in the ds*TaMet* experimental group were unobstructed, with some of the epithelial cells showing an irregular distribution (Figure 2E).

### 3.3 *TaMet* knockdown resulted in decreased reproductive ability in females

**FIGURE 3:**
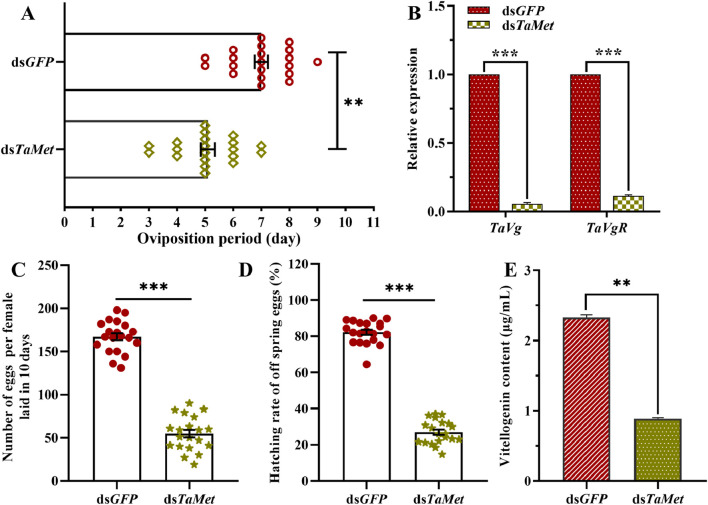
Effect of *TaMet* silencing on female reproduction in *Tuta absoluta.* **(A)** Oviposition period of females after dsRNA injection. **(B)** Expression levels of *Vg* and *VgR* after dsRNA injection. **(C)** Total number of eggs laid per female in 10 days **(D)** The hatch rate of offspring eggs. **(E)** Vitellogenin content in 2-day-old females after dsRNA injection. Significant differences between the treatment and control groups were determined using a Student’s *t*-test (*p < 0.05, **p < 0.01, ***p < 0.001).

Suppressing *TaMet* expression significantly decreased the female moths’ oviposition calendar period, which was reduced to 5.1 days compared to 7 days in the control group (Figure 3A). *TaVg* and *TaVgR* in *T. absoluta* significantly decreased after injection with ds*TaMet* (Figure 3B) (*t*_*vg*_ = 196.892, *t*_*VgR*_ = 60.432, *p* < 0.001). Females in the ds*TaMet*-injected group average laid 54.70 eggs per individual, which was significantly less than the 167.05 eggs per individual laid in the control group (Figure 3C) (*t* = 18.508, *p* < 0.001). The hatching rate of progeny in the ds*TaMet*-treated group was only 27%, which was significantly less than the 82.2% observed in the control group (Figure 3D) (*t* = 26.887, *p* < 0.001). The vitellogenin content was significantly lower (0.89 μg/mL) in 1-day-old *T. absoluta* females than in the control group (2.33 μg/mL; *t* = 60.432; *p* < 0.001) (Figure 3E).

### 3.4 *TaMet* knockdown resulted in molt failure and wing deformity

**FIGURE 4:**
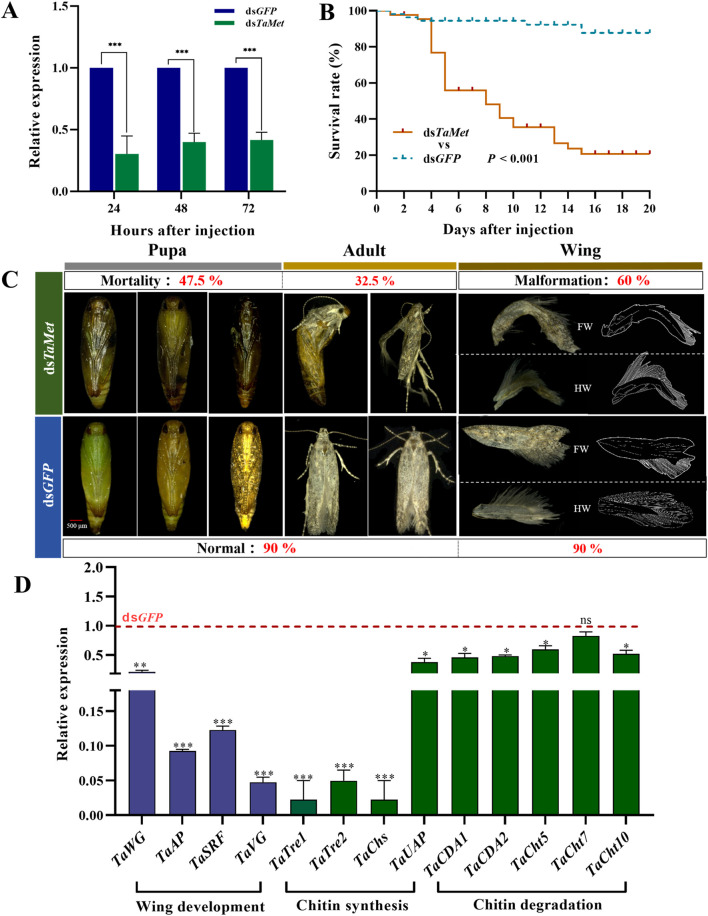
Effect of *TaMet* silencing in male *Tuta absoluta*. **(A)** Relative expression levels of *TaMet* at 24, 48, and 72 h after *TaMet* or *GFP* dsRNA injection. **(B)** Survival rate of males within 20 days of dsRNA injection. **(C)** Analysis of death phenotypes and wing deformity in males after dsRNA injection. FW: Forewings; HW: Hind wings. **(D)** The expression levels of genes involved in wing development, chitin synthesis, and degradation after dsRNA injection. Significant differences between the treatment and control groups were determined using a Student’s t*-*test (*p < 0.05, **p < 0.01, ***p < 0.001).

The expression level of *TaMet* was significantly reduced after injection with dsRNA at 24, 48, and 72 h (Figure 4A) (*t*_24h_ = 8.353; *t*_48h_ = 14.349; *t*_72h_ = 16.218; *p* < 0.001). The ds*TaMet*-injected group had only a 20% male survival rate after 20 days compared to 85% in the ds*GFP* group (Figure 4B). Statistical observations revealed that silencing the *TaMet* gene resulted in 47.5% of deaths during the pupal stage due to abnormal pigmentation or the inability to molt pupal sheaths, and 32.5% of deaths during the adult stage. Males injected with ds*TaMet* had a 60% chance of developing wing deformities in adulthood. Both the forewing and hindwing veins displayed varying degrees of atrophy and notching (Figure 4C). Silencing *TaMet* in males led to a significant reduction in wing development genes *TaWG* and *TaVG* by 76.8% and 95.6%, respectively. The expression of wing primordial formation genes *TaAP* and *TaSRF* was reduced by 90.6% and 87.1%, respectively. The expression of eight genes associated with chitin degradation and synthesis were variably suppressed (Figure 4D), suggesting that *TaMet* regulates chitin degradation and synthesis genes during the pupal–adult transition.

## 4 Discussion

The expression of hormone receptor genes at different insect development stages is regulated by JH (Yan et al., 2021). Specifically, during the transition from the nymphal to adult stages in the wheat red sucker, *SmMet* expression was significantly lower in females than in males, indicating that *SmMet* may play a crucial role in sustaining the effects in insects (Cheng et al., 2020). Elevated expression of the Met gene during the early pupation stages facilitates tissue reconstruction (Tettamanti and Casartelli, 2019). *HaMet* was predominantly expressed in the pupal and egg stages in *Harmonia axyridis* (Han et al., 2022). In this study, the *TaMet* expression pattern exhibited cyclical variations across developmental stages. In *T. absoluta, TaMet* expression was significantly higher in the early pupal and adult stages, which are critical periods for pupal growth and adult reproduction.

Insects exhibit a range of notable characteristics, particularly their pronounced reproductive and migratory capabilities. Consequently, our research concentrated on examining *TaMet* expression within the reproductive system and during the wing formation process in *T. absoluta*. *TaMet* expression in the adult ovary was significantly higher than in the spermatophore. *HaMet* expression in *H. armigera* was significantly higher in the ovary than in other tissues, including seminal vesicles (Ma et al., 2018). *TaMet* expression in *T. absoluta* was higher in the wings of males than in females during the same developmental period. These results are consistent with previous studies. *BmMet2* was highly expressed in the wing primordia of *B. mori* larvae and pupae (Kayukawa et al., 2012). Thus, it can be concluded that *TaMet* plays dual roles in reproduction and wing development.

In most female insects, Vg synthesis in fat bodies and oocyte maturation are regulated by JH (Luo et al., 2021; Liu et al., 2019). To investigate the function of *TaMet* in *T. absoluta* reproduction, we suppressed its expression using RNAi, and egg production, egg hatchability, ovary tube length, and egg granule length decreased as a result. We also noted a delay in ovary development. *SgMet* deficiency in *Schistocerca gregaria* results in delayed ovarian development and reduced female reproductive capacity (Gijbels et al., 2019). *SfMet* knockdown in *Sogatella furcifera* significantly reduces *SfVg* expression and yolk protein deposition and impairs oocyte maturation and ovarian development (Hu et al., 2019). Thus, *TaMet* is integral to female reproduction in *T. absoluta*. Furthermore, females injected with ds*TaMet* showed a decreased vitellogenin content and reduced *TaVg* and *TaVgR* gene expression, similar to the results for *TcMet* in *T*. *castaneum* (Naruse et al., 2020). Although silencing *RpMet* does not affect female egg production in *Rhodnius prolixus*, it reduces the hatching ratio (Leyria et al., 2022). Therefore, it is hypothesized that *TaMet* plays a crucial role in oocyte development and yolk formation. After gene silencing, the test insects were further stained using phenol magenta, showing that *TaMet* injection caused a loss of normal morphology in follicular epithelial cells and increased the cell gap. JH regulates vitellogenesis and oogenesis in insects via Met (Gujar and Palli, 2016). Met acts on *Mcm4* and *Mcm7* to regulate DNA replication and polyploidy for vitellogenesis and oocyte maturation (Guo et al., 2014). Similar results have been observed in *S*. *gregaria* (Holtof et al., 2021). Therefore, the decrease in *TaMet* expression may affect follicular epithelial cell development and yolk deposition, ultimately leading to ovarian morphological abnormalities and decreased fertility.

A decrease in the JH titer and an increase in the 20E titer at the end of the larval and pupal stages promote the completion of metamorphosis (Huang et al., 2013). JH suppresses the expression of the *laccase2* gene during embryonic development in *Blattella germanica*, preventing premature hardening and cuticle darkening (Fernandez and Belles, 2017). Similarly, reducing *AaMet* expression in *A. aegypti* results in mortality during the pupal stage of the offspring (Zhu et al., 2019). In this study, *TaMet* knockdown in male pupae led to significant mortality. During the transformation of pupae into adults, most ds*TaMet*-treated pupae exhibited blackening, abnormal molting, and death. We propose that *TaMet* suppression influences the inhibitory effect of JH on the *laccase2* gene, consequently resulting in the melanization and mortality of male *T. absoluta* pupae. During insect molting, abnormal regulation of stratum corneum formation can be fatal. Thus, the expression levels of genes involved in chitin metabolism are crucial for the degradation of the old epidermis and the synthesis of the new stratum corneum (Liu et al., 2022; Zhao et al., 2019). We found that silencing *TaMet* in male pupae resulted in significant reductions in the expression of genes critical for wing development and those involved in chitin metabolism. Depletion of *hormone receptor three* gene, another typical transcription factor in *Lasioderma serricorne*, disrupted the larval–pupal molting and downregulated the expression of chitin synthesis and degradation genes (Ma et al., 2022). These results suggest that different transcription factors are involved in the regulation of chitin metabolism during the insect molting and metamorphosis. In the larval stage of *Leptinotarsa decemlineata*, silencing of *LdMet* led to an increase in the 20E titer, resulting in shorter larval stages and early pupal weight loss (Meng et al., 2018). The effect of JH on insect metamorphosis through the “MEKRE93” pathway has been demonstrated in *B. germanica* (Belles and Santos, 2014). Additionally, Met knockdown also resulted in a reduction of insulin-like peptide (ILP) expression (Sheng et al., 2011). Therefore, we propose that *TaMet* knockdown in male *T. absoluta* pupae leads to plumage failure, possibly due to an increase in the 20E titer and insufficient energy reserves.

In the present study, *TaMet* showed functional differentiation in tomato leafminer moths of different sexes. Similarly, in *B. mori, BmMet1* is required for JH antagonism of larval metamorphosis, while *BmMet2* is involved in JH regulation of adult reproduction (Kayukawa and Shinoda, 2015). Only one *TaMet* gene has been identified in *T. absoluta*. It has become common for the same gene to exhibit functional differentiation in insects. Pleiotropy, which plays an important role in many organisms, refers to the phenomenon in which one gene determines the formation of multiple characteristics (Horabin and Schedl, 1993). In *D*. *melanogaster,* the *α1,4-galactosyltransferase one* gene has dual roles in spermatogenesis, including maintaining the survival of sperm bundles and regulating the sperm individualization process (Xiao et al., 2024). However, the gender-based functional differentiation of genes has rarely been reported in insects. In this study, *TaMet* mediated the male molting and female reproduction processes. Similar results have been found for the *triacylglycerol lipase* gene in *Sitotroga cerealella,* which regulates the sperm number in male moths and egg production in females (Yan et al., 2022). At present, there are few reports on the functional differentiation of the same gene in male and female insects, necessitating further research. Therefore, this study provides important materials for subsequent research on gene functional differentiation. We believe that *TaMet* is essential for the reproductive ability of *T. absoluta* females. *TaMet* mediates the male molting and wing development processes, making it a new potential target for RNAi-based control of *T. absoluta*.

## Data Availability

The datasets presented in this study can be found in online repositories. The names of the repository/repositories and accession number(s) can be found in the article/Supplementary Material.
