# Supplementary material for: Dual roles of methoprene-tolerant gene TaMet in male molting and female reproduction of the tomato leafminer, Tuta absoluta (meyrick)
Source: Front Physiol. 2024 Nov 14;15:1500391. doi: 10.3389/fphys.2024.1500391 (PMC11603827; doi:10.3389/fphys.2024.1500391)
Supplement: Supplementary file 1 [file Table1.docx]

Supplementary Material

# Supplementary Data

**Table S1. Primers used in this study.**

| **Gene name** | **Forward primer (5′-3′)** | **Reverse primer (5′-3′)** | **Application primers** |
| --- | --- | --- | --- |
| *TaMet* | GTCGTTTACAGCAAGATCAC | CCACATAGTTCTTCAGATGG | ORF confirmation |
| *TaMet* | GGCTGCTTTTCATGAGGACA | TGACCCAACGGACATCATCT | qPCR analysis |
| *TaEF1α* | CCTGGGCACAGAGATTTCAT | GATCAGCTGCTTGACACCAA |  |
| *TaVg* | TGGTACGTGGTTATGCAGGA | TACTTCGACACTGGGGGTTC |  |
| *TaVgR* | GCAAACCTGAGAGCCACTTC | CAGCGGCTGTCATTTCTACA |  |
| *TaSRF* | CCAACCCAACGATCCAACAG | AGTCCCAGGTTCGATCCAAG |  |
| *TaWG* | CAAGTGTCACGGCATGTCAG | ACTTGTGGTCGGGGTTGTG |  |
| *TaAP* | ACTTCGCGATCAACCACAAC | GAGCCCATCGGACCATGATA |  |
| *TaVG* | CAACGGATCCTCTGACTCGG | CAGGTTGCGAGACACCATC |  |
| *TaTre1* | GGTTAGACACGCAACGGATC | CTCCTGTTGCCGTTCTGATG |  |
| *TaTre2* | CGCGCCGATATTATTGAGGG | CTCCTTCCAAACACGTAGCG |  |
| *TaUAP* | AACCGCTCGAGATGATTTGC | ACCGTTACCATTCAGTCCGT |  |
| *TaChs* | TTGTCGTCCATGAGAGCCTT | CCTTGTGGCGGATCATGAAG |  |
| *TaCDA1* | AAGAAAGACGACAGCCTGGA | CTTCACTTTGCGCTCCTTGT |  |
| *TaCDA2* | TTCGTGCCACATTCTTCGTC | CCAATGATTGACGCATCGGT |  |
| *TaCht5* | AGAAGAAGGGCTATCTGGGC | AGGGTCAGGAGTTGTGGATG |  |
| *TaCht7* | CTCAGCGGGTACAAAGTTCG | GTGGTGCTTTCTCTCTCCCT |  |
| *TaCht10* | CCGGAAATGGATTAGGTGCG | CCTTGTGGCGGATCATGAAG |  |
| *TaMet* | **TAATACGACTCACTATAGGG**GGTAGACCAAGGTATGCTG | **TAATACGACTCACTATAGGG**ATTTGGGTCAAAGGTACT | dsRNA synthesis |
| *GFP* | **TAATACGACTCACTATAGGG**CAGTTCTTGTGAATTAGAT | **TAATACGACTCACTATAGGG**AATGTTACCATCTTCTTTAA |  |

*The sequence in bold at the 5’end of the primer is the T7 promoter sequence.
